# Closing the gender gap in medicine: the impact of a simulation-based confidence and negotiation course for women in graduate medical education

**DOI:** 10.1186/s12909-023-04170-y

**Published:** 2023-04-14

**Authors:** Anna Bona, Rami Ahmed, Lauren Falvo, Julie Welch, Melanie Heniff, Dylan Cooper, Elisa Sarmiento, Cherri Hobgood

**Affiliations:** 1 Department of Emergency Medicine, Indiana University School of Medicine, 720 Eskenazi Ave, Fifth Third Bank Building 3rd Fl, Indianapolis, IN 46202 USA; 2 Faculty, Division of Simulation, Department of Emergency Medicine, Indiana University School of Medicine, Indianapolis, IN USA; 3 Director of Emergency Medicine Simulation, Roudebush VA Medical Center, Indianapolis, IN USA

**Keywords:** Gender equity, Women in leadership, Simulation communication curriculum

## Abstract

**Background:**

Currently, 75–80% of the medical workforce worldwide consists of women. Yet, women comprise 21% of full professors and less than 20% of department chairs and medical school deans. Identified causes of gender disparities are multifactorial including work-life responsibilities, gender discrimination, sexual harassment, bias, lack of confidence, gender differences in negotiation and leadership emergence, and lack of mentorship, networking, and/or sponsorship. A promising intervention for the advancement of women faculty is the implementation of Career Development Programs (CDPs). Women physician CDP participants were shown to be promoted in rank at the same rate as men by year five, and more likely to remain in academics after eight years compared to both men and women counterparts. The objective of this pilot study is to investigate the effectiveness of a novel, simulation-based, single-day CDP curriculum for upper-level women physician trainees to teach communication skills identified as contributing to medicine’s gender advancement gap.

**Methods:**

This was a pilot, pre/post study performed in a simulation center implementing a curriculum developed to educate women physicians on 5 identified communication skills recognized to potentially reduce the gender gap. Pre- and post-intervention assessments included confidence surveys, cognitive questionnaires, and performance action checklists for five workplace scenarios. Assessment data were analyzed using scored medians and descriptive statistics, applying Wilcoxon test estimation to compare pre- versus post-curriculum intervention scores, with p < 0.05 considered statistically significant.

**Results:**

Eleven residents and fellows participated in the curriculum. Confidence, knowledge, and performance improved significantly after completion of the program. Pre-confidence: 28 (19.0–31.0); Post-confidence: 41 (35.0–47.0); p < 0.0001. Pre-knowledge: 9.0 (6.0–11.00); Post knowledge: 13.0 (11.0–15.0); p < 0.0001. Pre-performance: 35.0 (16.0–52.0); Post-performance: 46.0 (37–53.00); p < 0.0001.

**Conclusion:**

Overall, this study demonstrated the successful creation of a novel, condensed CDP curriculum based on 5 identified communication skills needed for women physician trainees. The post-curriculum assessment demonstrated improved confidence, knowledge, and performance. Ideally, all women medical trainees would have access to convenient, accessible, and affordable courses teaching these crucial communication skills to prepare them for careers in medicine to strive to reduce the gender gap.

**Supplementary Information:**

The online version contains supplementary material available at 10.1186/s12909-023-04170-y.

## Background

Worldwide, women make up 75–80% of healthcare workers and comprise 38% of academic faculty [1, 2]. However, women’s advancement in rank and into leadership roles decline significantly compared to men colleagues [1, 2]. Currently women comprise 21% of full professors and less than 20% of department chairs and medical school deans [1, 3]. The identified causes for these gender disparities are multifactorial, including work-life responsibilities, gender discrimination, sexual harassment, bias, lack of confidence, gender differences in negotiation and leadership emergence, and lack of mentorship, networking, and/or sponsorship [1, 4–8]. To counter or mitigate some of these factors, suggested solutions emphasize the importance of teaching women interviewing skills and negotiation techniques in addition to increasing women’s opportunities for sponsorship, mentorship, award recognition, speaking engagements, authorship, and editorial roles [5, 7, 8].

A promising intervention for the advancement of women faculty is the implementation of Career Development Programs (CDPs) [9]. Women physicians who participate in CDPs were shown to be promoted in rank at the same rate as men by year five, and more likely to remain in academics after eight years compared to both men and women counterparts [10, 11]. Further studies of CDPs reveal that women benefit with perceived improvement in communication, networking, and conflict management skills [12]. However, most development programs for women in medicine are offered after residency and fellowship training and are often expensive and time consuming.

Reviewing both the observed causes of the gender gap as well as potential solutions, there is a notable emphasis on the value of communication skills to aid in the retention and advancement of women in medicine [5, 12]. Simulation is a well-established methodology for teaching effective communication in healthcare [13, 14]. Additionally, several simulation-based curricula show successful leadership development in a variety of domains [15–19]. Since much of the identified need for women faculty is training on more effective communication strategies, simulation offers an optimal and underutilized approach to teach these necessary interpersonal communication skills [5, 12–14]. The objective of this pilot study is to investigate the effectiveness of a novel, simulation-based, single-day CDP curriculum for upper-level women physician trainees to teach communication skills identified as contributing to medicine’s gender advancement gap.

## Methods

To create this novel curriculum, we applied Kern’s Six-step approach to curriculum development as our overarching conceptual framework [20].

### Problem identification and general needs assessment

We identified the problem of gender disparities in the retention and promotion of women in medicine. The general needs assessment included both a literature review and interviews with men and women physicians and faculty.

The literature review included searches in medical, business, psychiatry and psychology academic journals and databases. Search terms included leadership, working women, leadership courses, AAMC data resources, women in medicine, women in leadership, and terms (“Leadership“[Mesh]) AND ((“Women, Working“[Mesh]) OR “Women“[Mesh])’ in PubMed.

One woman physician (AB) initiated all interviews via electronic mail solicitation. The interviewer conducted each interview via electronic mail correspondence, telephone call, or in-person. Semi structured interviews were focused on soliciting feedback on knowledge and experiences leading to gender disparity and potential solutions to close identified gaps. A total of 21 physicians and/or educators were included, comprising MDs, DOs, MD/JDs, MD/MBAs, and PhDs serving as adjunct, assistant, associate, and full professors and ranging from less than one year to greater than 15 years out from residency or doctoral training.

### Targeted needs assessment

Literature findings and interview responses were collated and reviewed to identify recurrent and pervasive themes. The study group, consisting of content experts in education and simulation, evaluated the identified themes to develop goals and objectives for teachable skills conducive to a simulation-based curriculum. The four major communication skills identified were: communicating an effective elevator pitch, successfully self-promoting, contract negotiation, and conflict de-escalation.

### Goals and objectives

Based on the identified skill gaps from the needs assessment, we created four formative simulation scenarios with specific goals and objectives described by specific skills performance checklists. We collated those four checklists to form one cohesive pre/post scenario simulation action checklist [Appendix 1]. We additionally created a summative scenario to apply the cohesive pre/post scenario action checklist to evaluate participants’ skills before and after the four formative scenarios [Appendix 2].

We solicited feedback on the scenarios and checklists from the initial 21 interviewees. From their feedback, we recognized the need to add the skill of opening/closing a meeting. The opening/closing of a meeting skill was subsequently included in the formative and summative simulation scenarios and checklists. All five communication skill gaps were considered when developing confidence assessment, knowledge multiple choice questions (MCQs), and skills performance checklists tools.

The cases and checklists were then distributed a second time via electronic mail to each of the initial interviewees for review. Feedback was provided by 12 of the initial 21 interviewees. This feedback was again collected via electronic mail, telephone call, or in-person. The project authors conducted final edits for the cases and data collection instruments.

### Educational strategies

Once the four educational formative and the single cohesive pre/post summative simulation cases and checklists were finalized, we designed the remaining program to teach the 5 communication skills of opening/closing a meeting, communicating an effective elevator pitch, successfully self-promoting, contract negotiation, and conflict de-escalation (available upon request to corresponding author). The conceptual frameworks utilized within the overarching curricular framework of Kern were Adult Learning Theory, Vygotsky’s Sociocultural Theory, Kolb’s Experiential Learning Style Theory (KELST), Behavioral Learning Theory, and Rapid Cycle Deliberate Practice (RCDP). [21–25].

Adult Learning Theory principles guided the creation of simulation scenarios with topics relevant to participants’ current or immediate stages of their careers. Problem centered simulation scenarios utilized the single overarching topic of patient safety as the consistent focal discussion point for the educational formative and pre/post summative simulations. Simulations provided an experience for the participants to practice, while making and learning from mistakes. At the course’s conclusion, participants were invited to give evaluative feedback [21].

Applying Sociocultural Theory, the content lectures and checklists provided scaffolding for participants. Scaffolding provides a temporary framework for participants to utilize as they progress towards independent problem-solving and skills competence [22]. The scaffolding of the lectures and checklists is not intended to be an exhaustive, comprehensive approach to the 5 communication skills. They do, however, provide feasible learning objectives targeted at the learner’s zone of proximal development [22]. The course structure was iterative and placed more complex learning objectives and skills in the afternoon session, building upon the concepts taught and practiced in the morning’s session. Women faculty served as the theory’s “more knowledgeable other” and led the debriefings [22].

Consistent with KELST, the simulations served as concrete experiences. Debriefing promoted reflective observation, allowing for abstract conceptualization for participants to study and modify their prior concepts [23]. Employing Behavioral Learning Theory, standardized patients (SPs) trained in the role of the Department Chair provided direct interactive feedback during the simulated scenarios. Course faculty provided coaching, in either a RCDP format, or a standard simulation and debrief followed by a deliberate practice repeat simulation and debrief [24, 25]. In addition to providing new concrete experiences, the curriculum’s repetitive simulations allowed for active experimentation providing participants the opportunity to incorporate coaching feedback and apply new concepts and skills [23, 24].

### Implementation

Participant recruitment was conducted via electronic mail and submission on a Google Form. Participants included any woman upper-level resident or fellow, in the final 2 years of training at Indiana University School of Medicine. Electronic mails were sent to chief residents, program directors, and program coordinators for distribution to residency and fellowship training programs in central Indiana. The curriculum was provided at The Simulation Center at Fairbanks Hall, which supports the education of IU Health and Indiana University Schools of Medicine and Nursing. Recruitment was a challenge as invitations were sent eight weeks in advance with many residents or fellows having established call and clinic schedules with 65–80-hour work weeks and limited time off to participate in such pilot curricula. Video of the pre and post-test simulations were obtained via iPad recording on stands in each room.

A total of 11 women participated in this pilot study curriculum, with five on the first day and six on the second. The participant characteristics are elaborated in (Table 1). Participants were provided informed consent including videorecording, risks, and benefits of the study. This study was approved exempt by the Indiana University institutional review board (IU IRB #1,910,529,789).

**Table 1:** Participant Characteristics, n = 11

Mean age (range)	29.1 (26–32) years
Mean Post-Graduate Training Year (range)	3.27 (2–4)
Training Role	Resident: 8 Fellow: 3
Specialties	IM 3. Med-Peds 1. ID 1. EM 4. Peds 2.
Prior Leadership and/or Business Course(s) Participants	1

Legend: IM: Internal Medicine; Med-Peds: Internal Medicine-Pediatrics combined residency; ID: Infectious Disease; EM: Emergency Medicine; Peds: Pediatrics

This single day curriculum was conducted on two separate dates. The overall curriculum consisted of two in-person lectures given by the lead author (AB) and four formative simulation scenarios covering the skills of communicating an effective elevator pitch, successfully self-promoting, contract negotiation, and conflict de-escalation (Table 2). Each scenario included content on opening/closing a meeting. The course structure allowed a maximum of 8 participants per date. The described content was separated into a part one (morning) and part two (afternoon curriculum).

The participants started with the pre-test summative simulation case, and then completed a 15-item multiple-choice questionnaire, and a 10-item confidence survey.

Part one’s curriculum included a 60-minute interactive didactic lecture, followed by the elevator pitch and self-promotion educational formative simulations. Part two of the curriculum consisted of a second, 60-minute content lecture and formative simulations on contract negotiation and conflict de-escalation. The content lectures incorporated educational material expanding on proposed tools and solutions identified in the background and literature needs assessment searches and applying the checklists as scaffold tools. This included background and contributing factors on gender inequity, highlighting promotion, retention, and pay. It included business literature, primarily focused on communication techniques for effective elevator pitches, contract negotiation, and psychological studies and theories that identify social norms and other biases affecting communication with tools to address these barriers (available upon request to corresponding author). The lecturer’s techniques and theories were the basis of the assessment tools of utilized to evaluate the participants confidence, knowledge, and performance skills.

After the curriculum intervention, the day concluded with the summative simulation post-test case. This case was followed by the identical multiple-choice questionnaire, confidence survey, and a course evaluation and feedback form. The same three independent evaluators scored video recordings of participant performance in the summative case with the checklist. Please see Table 2 for a schedule of day.

**Table 2:** Curriculum Schedule

Time	Morning	Time	Afternoon
8:00–8:30 AM	Introductions and Pre-brief	12:15–2:00 PM	Lecture II, Lunch
8:30–9:00 AM	Pre-test Simulation, then MCQ’s and Confidence Pre-test	2:00–3:00 PM	Case 4/5 Simulation
9:00–10:15 AM	Lecture I, Break	3:00–4:00 PM	Case 5/4 Simulation
10:15–11:15 AM	Case 2/3 Simulation	4:00–4:30 PM	Post-test Simulation, then MCQ’s, Confidence Post-test, Post-course evaluation and feedback form
11:15 AM – 12:15 PM	Case 3/2 Simulation	4:30–5:00 PM	Group Debrief, Wrap up

Legend: Pre/Post Test Simulation: Summative Case 1/6; Case 2: Communicating an Elevator Pitch; Case 3: Self-promotion; Case 4: Contract Negotiation; Case 5: Conflict De-escalation; MCQ’s: Multiple-Choice Questions

The identical pre- and post-summative case was conducted one-on-one with the participant and a standardized patient Department Chair and video recorded for assessment. For consistency of data collection, the same three individuals served as the SP department chair for the pre- and post-test case on both dates. These individuals were selected given their extensive job experience in simulation. For the formative simulations, the standardized Department Chair also included volunteer faculty physicians. The SP Department Chairs underwent a 4-hour in-person training session led by the lead author. Training included reviewing the SP materials, discussing the participants’ goals and objectives, and practice rehearsing the cases. All standardized Department Chairs were white men, over the age of forty years. All faculty educators were women physicians who spanned all academic ranks.

For the four formative cases, two were conducted in the standard simulation format and the other two were conducted using the RCDP format. Each curricular component had one case in each style. The self-promotion and contract negotiation cases were conducted using a standard single case simulation format, followed by a debrief. This involved 1 participant, with 1 SP Department Chair, and 1 faculty. Given the compact single-day curriculum and the allotted one-hour scheduled, this structure allowed for an additional deliberate practice repeat of the educational scenario, followed by a second debrief. The elevator pitch and conflict de-escalation cases were conducted using RCDP. The RCDP cases were designed for 2–4 simultaneous participants, 1 SP Department Chair, and 1 faculty. RCDP simulation method has a faculty educator present throughout the simulation scenario to practice “within-event” micro-debriefing (pause, debrief, rewind, and try again) for deliberate practice. This method is ideal for skills that have defined steps, for which we applied the course’s formative case checklists. We selected this format as it has been shown to allow for early error correction and to improve participants’ skills and confidence [25]. In the RCDP format, we also rotated participants in the active simulation role, allowing peers an opportunity to observe another participant’s approach and learn with and from each other throughout the scenario’s micro-debriefs. The course’s case structure with the simulation format, number of participants, faculty, and SPs is outlined in (Table 3).

**Table 3:** Curriculum Structure

Case	Name	Format	Number of Participants	Number of SP	Number of Faculty
Pre-test 1	Summative Simulation	Standard, no debrief	1	1	0
Case 2	Communicating an Elevator Pitch	RCDP	2–3	1	1
Case 3	Self-promotion	Standard, repeated twice	1	1	1
Case 4	Contract Negotiation	Standard, repeated twice	1	1	1
Case 5	Conflict De-escalation	RCDP	2–3	1	1
Post-test 6	Summative Simulation	Standard, no debrief	1	1	0

Legend: SP: Standardized patient Department Chair; RCDP: Rapid Cycle Deliberate Practice

### Evaluation and feedback

We evaluated participants’ confidence, knowledge, and performance pre- and post-curriculum intervention for the 5 stated subcategories. Customized evaluation tools were developed by our study team using a modified Delphi method. A lack of available tools in the literature necessitated this development. Participant confidence was assessed using 10 items self-rated on a 5-point Likert scale. Fifteen multiple-choice questions designed and piloted on recent women graduates assessed the participants’ knowledge domain.

Three independent evaluators scored participant skills performance of the summative case with the 62-item checklist. Each case was scored by two evaluators. Evaluators were blinded from the knowledge if the videos they were assigned to review were pre or post intervention. The evaluators scored the skills checklist as 0.0 for not completed/performed incorrectly or 1.0 for performed correctly.

The course concluded with a 9 item post-course evaluation form. We collected performance data only on the confidence survey, MCQs, and pre/post summative simulation case checklist. The four formative simulations were solely for educational purposes.

### Pre- and post-evaluation and analysis

Pre- and post-test simulation scenario videos were reviewed by three independent author evaluators. Evaluators were trained by the lead author in two sessions, scheduled one week apart. The first 3-hour session reviewed the summative case checklist, created the grading rubric through expert consensus, and discussed its application to one simulation scenario. Each evaluator then individually reviewed and scored three different scenarios in preparation for the second session. The second 2-hour session was designed to improve inter-rate reliability by reviewing the application of the rubric to additional simulation scenarios. The evaluators were advised to review all videos in two or more sessions to avoid evaluator fatigue and to employ the rubric for consistency. The scenario videos were de-identified of pre- versus post-test scenario.

We used descriptive statistics to calculate before and after curriculum intervention scores for the summative checklist and its 5 subsets in confidence survey, knowledge MCQs, and skills performance assessment. We used Wilcoxon test to describe the change in all scores after training. Interrater reliability between the evaluators was estimated using the simple kappa coefficient. All statistical analyses were performed using SAS Version 9.4.

## Results

Results demonstrated overall improvement in confidence, knowledge, and performance post-curriculum intervention.

For the summative checklist, we observed improvement in each of the 5 subsets, as well as the overall performance (Table 4). Evaluator interrater reliability was fair to moderate (Table 5) [26]. Knowledge of the skills, as assessed by the multiple-choice questions, improved significantly overall as well as in the opening/closing, self-promotion, and conflict de-escalation subcategories (Table 6). Confidence scores improved in all subcategories, as well as overall (Table 7).

**Table 4:** Skills Performance Checklist Scores, Median (Min-Max)

	Pre	Post	P-Value*
**Categories**			
Opening/Closing	6.0 (1.0–9.0)	8.0 (6.0–9.0)	**< 0.0001**
Elevator Pitch	13.0 (5.0–15.0)	16.0 (10.0–18.0)	**< 0.0001**
Self-Promotion	3.0 (1.0–6.0)	5.0 (1.0–6.0)	**0.0022**
Negotiation	3.0 (1.0–6.0)	5.0 (30 − 7.0)	**0.0002**
Conflict De-escalation	10.0 (3.0–17.0)	14.0 (9.0–17.0)	**< 0.0001**
**Total**	35.0 (16.0–52.0)	46.0 (37.0–53.0)	**< 0.0001**

*Estimated using Wilcoxon test

**Table 5:** Skills Performance Evaluator Interrater Reliability

Raters	Estimate	95% Confidence Limits
Rater 1/ Rater 2	0.59	0.55–0.64
Rater 2 / Rater 3	0.47	0.41–0.52
Rater 1 / Rater 3	0.48	0.43–0.53

*Estimated using Simple Kappa Coefficient

**Table 6:** Knowledge Multiple-Choice Questions Scores, Median (Min-Max)

	Pre	Post	P-Value*
**Categories**			
Opening/Closing	2.0 (1.0–2.0)	2.0 (2.0–2.0)	**0.0147**
Elevator Pitch	2.0 (0.0–3.0)	2.0 (1.0–3.0)	0.3546
Self-Promotion	1.0 (0.0–2.0)	3.0 (2.0–3.0)	**< 0.0001**
Negotiation	3.0 (1.0–3.0)	3.0 (2.0–3.0)	0.5949
Conflict De-escalation	2.0 (1.0–3.0)	3.0 (2.0–4.0)	**0.0156**
**Total**	9.0 (6.0–11.0)	13.0 (11.0–15.0)	**< 0.0001**

*Estimated using Wilcoxon test

**Table 7:** Confidence Scores, Median (Min-Max)

	Pre	Post	P-Value*
**Categories**			
Opening/Closing	6.0 (5.0–8.0)	9.0 (8.0–10.0)	**< 0.0001**
Elevator Pitch	6.0 (4.0–8.0)	8.0 (7.0–10.0)	**0.0006**
Self-Promotion	6.0 (4.0–8.0)	8.0 (7.0–10.0)	**0.0007**
Negotiation	4.0 (2.0–7.0)	8.0 (6.0–10.0)	**0.0002**
Conflict De-escalation	5.0 (3.0–6.0)	7.0 (6.0–9.0)	**< 0.0001**
**Total**	28.0 (19.0–31.0)	41.0 (35.0–47.0)	**< 0.0001**

*Estimated using Wilcoxon test

## Discussion

Utilizing Kern’s Six-step approach to curriculum development, we successfully created and piloted a novel, single day simulation-based career development program curriculum for women physician trainees. Study participants demonstrated a statistically significant improvements in communication skills confidence, knowledge, and performance for the 5 communication skills of opening/closing a meeting, communicating an effective elevator pitch, successfully self-promoting, contract negotiation, and conflict de-escalation.

This curriculum makes a significant contribution to the career development literature because it uses a simulation-based curriculum to teach gender specific leadership skills. The majority of existing career development programs are of longer duration, focus on content delivery rather than skill development exercises, and require a competitive application process [10, 11]. This novel curriculum can be deployed in one day and is an efficient and effective method for gaining needed leadership communication skills, making it more readily available to the target audience prior to women entering the workforce.

In alignment with other work, we have demonstrated that use of a simulation-based curriculum is effective in teaching communication skills [12–14]. We expand upon this literature by our focus on gender specific leadership communication. Simulation educational theories served as the foundation for the educational strategies applied in the course curriculum. The intentional application of learning theories to the course design fostered repetitive skill crossover. All course faculty were women. This was done to apply Sociocultural Theory’s “more knowledgeable other’s” feedback. Additionally, this fostered professional relationships to address the identified lack of mentorship and sponsorship for women. The standardized patient Department Chairs were white, middle-aged men to reflect the current academic medicine leadership environment [27]. The use of detailed, nuanced checklists provided participants with a scaffolding framework, which both guided their actions and provided a reference for feedback, all of which fortify and inculcate the learning.

Despite the small data size, confidence scores improved markedly in all categories. The statistical significance of each subset is striking, as there were only 2 statements per subset that participants self-graded on Likert scales from 1 to 5. The statistical improvement in each subset is likely attributed to the curriculum’s extensive needs assessment and rigorous design, with clear lecture material and focused, formative simulations. The notable confidence improvement suggests a lack of prior education or familiarity with these communication tools in the current physician education training. Knowledge, assessed by the multiple-choice questions, improved significantly overall. Each subset only had 2–4 questions, limiting the sample size. Despite the participants having strong test-taking skills given their medical education, we still found subset improvement in the opening/closing, self-promotion, and conflict de-escalation subcategories of this assessment. Knowledge improvement we credit to the high- quality lectures and participants’ application in simulation practice. These improvements may be partially attributed to a selection bias of participants who voluntarily self-selected to participate and were personally motivated to improve prior to entering the workforce. Although not assessed in this study, future studies should assess long-term retention of the skills acquired.

The globally improved assessment results suggest the curriculum’s content was appropriate and effective. This is noteworthy given the volume of novel educational content presented in such a short interval over a single day. The study’s findings emphasize the identified need to incorporate this curriculum content into medical education. Additional future directions include course adaption for early women faculty, course development for other minorities in medicine, and a follow up of course participants to determine if participation resulted in earlier career promotion and leadership advancement.

The most notable study limitation is the small sample size of 11 participants. Future studies with larger samples should be performed. Providing a greater lead time for invitation to participate in the curriculum as well as discussing participation with program leadership prior to a new academic year to ensure trainees have time set aside to participate will help with future recruiting efforts. The development and utilization of non-validated tools also limits generalizability. The fair to moderate interrater reliability was also a significant limitation. Use of the 62-item assessment is time consuming and limits reproducibility. Shortening the performance checklist to reduce evaluator decision fatigue may improve this limitation in future iterations. In addition, the use of non-expert evaluators to assess performance in the future will reduce pre-existing bias. All participants were from a single institution which may limit generalizability; however, it is unlikely that gender specific leadership skills differ between institutions. Ideal next steps include having this study executed at multiple institutions.

## Conclusions

Overall, this study demonstrated the successful creation of a novel, condensed career development program curriculum based on 5 identified communication skills needed for women physician trainees. The post- curriculum assessment demonstrated improved confidence, knowledge, and performance. Ideally, all women medical trainees would have access to convenient, accessible, and affordable courses teaching these crucial communication skills to prepare them for careers in medicine and reduce the gender gap.

## Electronic supplementary material

Below is the link to the electronic supplementary material.

Supplementary Material 1

Supplementary Material 2

## Data Availability

The datasets used and/or analyzed during the current study are available from the corresponding author on reasonable request.

## List of abbreviations

CDP: Career Development Program
KELST: Kolb’s Experiential Learning Style Theory
RCDP: Rapid Cycle Deliberate Practice
IM: Internal Medicine
Med-Peds: Internal Medicine-Pediatrics combined residency
ID: Infectious Disease
EM: Emergency Medicine
Peds: Pediatrics
MCQ: Multiple-Choice Questionnaire
SP: Standardized patient
