# Supplementary material for: Closing the gender gap in medicine: the impact of a simulation-based confidence and negotiation course for women in graduate medical education
Source: BMC Med Educ. 2023 Apr 14;23:243. doi: 10.1186/s12909-023-04170-y (PMC10103407; doi:10.1186/s12909-023-04170-y)
Supplement: Supplementary file 1 — Supplementary Material 1 [file 12909_2023_4170_MOESM1_ESM.docx]

**Pre/Post Case (Case 1 AND 6)**

| **Phase 1: Establishing Rapport** | | YES | NO | Deficient |
| --- | --- | --- | --- | --- |
| 1. | Greets meeting participant(s) individually by name and title. |  |  |  |
| 2. | Introduces self briefly (and includes relevant title if existent). |  |  |  |
| 3. | Is confident and warm. Makes eye contact and shakes hand with a welcoming facial expression. |  |  |  |
| 4. | States any greeting after introduction, which may include mild flattery. (How are you? Nice to see you. I am honored to meet you. Thank you for meeting me.) |  |  |  |
| **Phase 2: Elevator Pitch, Engaging Your Audience** | | YES | NO | Deficient |
| 5. | Uses a “hook:” A brief initial preview statement/question that should engage and catch the listener’s interest. |  |  |  |
| 6. | The hook is brief, <10 seconds. |  |  |  |
| 7. | The hook is jargon free. |  |  |  |
| 8. | The hook is applicable to the target audience. (Appropriate department, individual, etc.) |  |  |  |
| 9. | Conveys a clear objective for the target audience. |  |  |  |
| 10. | Performs a knowledge assessment of the audience. |  |  |  |
| **Phase: 3: Elevator Pitch, Communicating the Proposal and Request** | | YES | NO | Deficient |
| 11. | Explicitly states the problem to solve. |  |  |  |
| 12. | Focuses on shared, beneficial goals: Patient safety, cost effectiveness, department/hospital benefits. |  |  |  |
| 13. | Explains “why” by emphasizing loss aversion: mortality, costs, etc. |  |  |  |
| 14. | Gives specific data to support “why.” |  |  |  |
| 15. | Focuses on the relevant broader perspective: patient, department, hospital, institution, nationally. |  |  |  |
| 16. | Proposes a solution to the problem. |  |  |  |
| 17. | Is authentic: Does not tell lies or misleading statements. |  |  |  |
| 18. | Is prepared and demonstrates knowledge about the proposed solution. |  |  |  |
| 19. | Shares if there is or is not proof of concept of the proposed solution being previously utilized successfully. |  |  |  |
| 20. | Is able to provide references appropriately. |  |  |  |
| 21. | Explicitly requests something to aid in achieving the proposed solution: meeting, feedback, point of contact, etc. |  |  |  |
| 22. | Pauses after explicit request. |  |  |  |
| 23. | Elicits questions. |  |  |  |
| **Phase 4 : Self Promotion, Declaring Qualifications for the Role** | | YES | NO | Deficient |
| 24. | Explicitly states that she qualifies for this role. |  |  |  |
| 25. | Contextualizes qualifications: States previous accomplishments, degrees, courses, positions, titles, lectures, and/or publications. |  |  |  |
| 26. | Uses concrete data: Evaluation forms, patient satisfaction scores, publications, success outcomes from prior projects. |  |  |  |
| 27. | Explains shared benefits: Success would reflect well on the department/hospital, etc. |  |  |  |
| 28. | Demonstrates confidence. |  |  |  |
| 29. | Allows for questions. |  |  |  |
| **Phase 5: Contract Negotiation, Developing and Negotiating Contract** | | YES | NO | Deficient |
| 30. | Clearly states the problem at hand, identifying the problem by name. |  |  |  |
| 31. | Asks the boss for their proposed funding. |  |  |  |
| 32. | States clearly the desired funding. |  |  |  |
| 33. | Pauses. The next individual to speak should be the boss. |  |  |  |
| 34. | Repeats the requested funding and provides supportive data, rationale, or justification for stated funding. |  |  |  |
| 35. | Asks about the boss’s perspective (not position), including requesting information or data for funding rationale. |  |  |  |
| 36. | Actively listens: pauses, nods, repeats phrases, summarizes points. |  |  |  |
| 37. | Avoids focusing on “bottom line” funding number, and instead tries to understand the counteroffer’s rationale using inquiries. |  |  |  |
| 38. | Tentatively agrees on funding.  -SP- Offer: 70% funded by ICU/30% by ED. This then leads to a conflict regarding funding between departments. |  |  |  |
| **Phase 6: De-escalation, understanding the Conflict - People & Interests** | | YES | NO | Deficient |
| 39. | States the current conflict. |  |  |  |
| 40. | Assures all parties that they are working side-by-side to determine and achieve the same goal. |  |  |  |
| 41. | Separates the people present from the present conflict. |  |  |  |
| 42. | Asks for and assesses each party’s priorities and objectives. |  |  |  |
| 43. | Asks why that is a priority. |  |  |  |
| 44. | Verbalizes empathy with all parties’ perspectives/frames. |  |  |  |
| 45. | Identifies and states common, shared interests of all/most involved parties, ideally by restating the party’s phrasing. |  |  |  |
| 46. | Avoids blame, assigning traits, offensive statements, or behavior that risks damaging relationships |  |  |  |
| 47. | Asks participants to identify and verbalize their emotions. |  |  |  |
| 48. | Repeats and validates empathetically the presented emotions for confirmation. |  |  |  |
| **Phase 7: Resolving Conflict - Options and Criteria** | | YES | NO | Deficient |
| 49. | Provides solutions with mutual gains. |  |  |  |
| 50. | Avoids positional bargaining statements. |  |  |  |
| 51. | Involves all parties as being invested creators in the solution. |  |  |  |
| 52. | Avoids emotional outbursts. |  |  |  |
| 53. | Focuses on the future. Re-directs any prior conflicts. |  |  |  |
| 54. | Emphasizes objective standards as the negotiation criteria. |  |  |  |
| 55. | Reframes any remaining conflict as an opportunity to search for a mutually beneficial solution. |  |  |  |
| 56. | Repeats steps as needed until a solution agreeable to all parties is established. |  |  |  |
| 57. | Answers any questions. |  |  |  |
| **Phase 8: Next Step(s) & Professional Closing** | | YES | NO | Deficient |
| 58. | Summarizes agreement |  |  |  |
| 59. | States the next steps. |  |  |  |
| 60. | Establishes follow up (meeting, email, etc.) including contact information exchange if needed. |  |  |  |
| 61 | Expresses gratitude. |  |  |  |
| 62. | Farewell Exchange. |  |  |  |
