# Supplementary material for: Closing the gender gap in medicine: the impact of a simulation-based confidence and negotiation course for women in graduate medical education
Source: BMC Med Educ. 2023 Apr 14;23:243. doi: 10.1186/s12909-023-04170-y (PMC10103407; doi:10.1186/s12909-023-04170-y)
Supplement: Supplementary file 2 — Supplementary Material 2 [file 12909_2023_4170_MOESM2_ESM.docx]

**Pre/Post Case (Case 1 AND 6)**

**Learner Door Note**

**Department Chair’s Name**: Dr. Blackwell

**Profile**: Hospital Division Chief of Patient Safety and Quality Control (PSQC). Manages each department’s needs as well as the hospital as a whole.

**Background**: Your employer, General Hospital, is having challenges with central line associated bloodstream infections (CLABSI).  You found a research article that highlighted the success of using simulation training for central venous catheter placement and the subsequent decreased rate of central line infections. In this article, the learners whom received simulation based medical education showed improved skills with decreased errors and increased placement success rates following the training. There was an 85% reduction in catheter-related bloodstream infections. Additional studies showed a return on investment of 7 to 1, meaning for every $1.00 applied towards the training, the hospital benefited $7.00.

**CLABSI metrics for General Hospital**:

- Rate: 15% above national average
- Patient outcomes: 12-25% mortality (CDC nation-wide metrics)
- Financial per incidence: $16,550 (CDC values) up to $74,000 (Agency for Healthcare Research and Quality) in excess healthcare costs incurred by the hospital

**Department’s priorities**:

- Patient safety
- Staff education
- Staff safety and wellness

**Budget Funding Obligations**:

- Grand Rounds speakers
- CME Accreditation annual fee
- Department’s Annual Staff Outing
- Research funding
- Staff conference attendance sponsorship

**Present Scenario**: Based on your research and prior experiences, you would like to propose the solution of implementing central line placement training using simulation based medical education. You have gathered information in preparation for proposing your solution. Training would cost approximately $100,000. There is currently no funding designated for these training resources.

**Anticipated Training Costs and Time proposal**:

- Estimated total cost: $100,000.00
- Training duration: 3 hours per session
- Minimum number of training sessions: 10
- Continued training costs anticipated at $3,000 annually

**Objective**: To propose your solution given the information above and negotiate with the PSQC Division Chair to obtain funding and resources.

**Pre/Post Case (Case 1 AND 6)**

**Training Material for the Standardized Patient**

**Role**: Dr. Blackwell, Hospital Division Chief of Patient Safety and Quality Control (PSQC). Manages each department’s needs as well as the hospital as a whole.

**Profile**:

1. Name: Dr. Blackwell
2. Age: 30-70
3. Gender: Male
4. Affect (mannerisms, behavior): Confident, Self-Assured.
5. Social: Healthy, Well-off
6. Occupation: Physician, Division Chief of Patient Safety and Quality Control (PSQC). Manages each department’s needs as well as the hospital as a whole.
7. General appearance: Business Professional (button up long sleeve, and may wear a tie or jacket), neat in appearance

**Scenario**:

You are the Hospital Division Chief of Patient Safety and Quality Control (PSQC) partaking in a board room meeting to discuss central line associated bloodstream infections (CLABSI). The learner wanted to share an idea to reduce central line associated bloodstream infections (CLABSI). You are aware that your employer, General Hospital, has CLABSI rates 15% above the national average

**Your Division’s priorities**:

- Patient safety
- Staff education
- Staff safety and wellness

**Division’s and Department’s Funding Obligations**:

- Grand Rounds speakers
- CME Accreditation annual fee
- Department’s Annual Staff Outing
- Research funding
- Staff conference attendance sponsorship

**Instructions for the Department Chair**:

1. **To begin the scenario**:
   1. If they do not initially state the purpose of the meeting, you should inquire, “So, what idea did you want to share with me today?”
   2. If they state the problem, but then stop, ask, “Why is this important?” or, “What is your idea?”
   3. You are aware of the hospital’s high CLABSI rate and interested in finding a solution, but hesitant to support the idea and give the funding costs and resources.
2. **Demeanor throughout the scenario**:
   1. Professional
   2. **PHASE 1**. Interested in the learner’s idea, but **hesitant** to support the idea without being convinced of its true value and effectiveness. Curious to know WHAT the idea is and WHY you should care.
   3. **PHASE 2.** After discussing the proposal for simulation education, you have **concerns and doubts** about the learner’s qualifications and capabilities.
   4. **PHASES 3 and 4.** Pause after hearing their initial request. Want to see data to support their requests. You are **offended** that the proposed idea of central line training is using so many resources and funding to teach your experienced physicians a skill that they already know. You are also **resistant** because even though you support patient safety, you don’t have this amount of money laying around. Your funding is allocated towards the division’s priorities.
3. **Concerns regarding the proposed solution**:
   1. **PHASE 1**. Hesitant to support because it is a substantial funding and resources request.
   2. **PHASE 2.** Doubts about the learner’s qualifications and capabilities for such a large and important task undertaking.
   3. **PHASES 3 and 4.** Offended being asked to teach experienced physicians a skill that they already know. Resistant because you have no control over the division’s fixed budget, and the current funding is already allocated towards the division’s priorities.
4. **Response to the learner’s statements and communication style**:
   1. **Continue expressing doubt, concerns, and questioning if the learner**:
      1. **PHASE 1:** Does not state the problem clearly. (CLABSI or central line infections). Example response statement: “What problem exactly did you want to discuss today?”
      2. Does not explain why this is a problem. Example response statement: “Why is this important? How does this help our patients or our hospital?”
      3. Fails to provide supportive data (mortality, hospital costs). Example response statement: “What information do you have to support this idea?”
      4. **PHASE 2:** Uses searching statements (I think/hope/wish/would like to be/etc., or asks for your opinion on their qualifications) instead of concrete statements (I am qualified…). This exactitude is similar to declaring someone has “died,” not “passed away.” Example response statement: “You think or hope you are qualified? But I want to know if you ARE qualified or not.”
      5. Does not explain their qualifications with references or data.
      6. **PHASES 3 and 4**: Does not state clearly their proposal or provide rationale for their proposal.
      7. Does not pause after stating their request.
      8. Does not try to understand your priorities and justification for your budget limitations. Example response statement: “Well, you don’t understand that I don’t just have this amount of money to give away. We have valuable financial obligations already that are important.”
      9. Fails to provide and explain solutions that are mutually beneficial. Example response statement: “How does this help the hospital out?”
      10. Assigns blame or questions the hospital’s capabilities. Example response statement: “So you think my hospital’s ICU physicians don’t know how to place a central line? This is OUR fault?”
      11. **GENERAL**: Is hesitant or unconfident. Example response statement: “Well, you don’t sound very confident in your proposed solution, so it is hard for me to feel confident about your idea.”
      12. Makes statements that end in tone with an upward inflection (that sound like a question instead of a statement). Example response statement: “Is that a question? Are you certain?”
   2. **Become more agreeable and supportive of the learner’s request if the learner***:

*You may still ask why, for supporting data, or if there was prior use of this solution.

- - 1. **PHASE 1**: States the problem clearly. (CLABSI or central line infections).
    2. Explains why this is an important problem and provides supportive data.
    3. Explains the solution to use central line simulation education.
    4. **PHASE 2:** Uses concrete statements that they are qualified for this role.
    5. **PHASES 3 and 4**:
    6. Assesses your initial offer before making their request.
    7. Pauses after making their request. WAITS for you to speak and respond first.
    8. Provides data to support their requests.
    9. Avoids focusing on “bottom line” positional negotiation. The learner does not focus on the *number*, but instead tries to *understand* your counteroffer’s rationale
    10. Clearly states a request: amount of money, a follow up meeting, point of contact, etc.
    11. Does not assign blame or question department capabilities.
    12. Recognizes your frustrations, shows empathy, and assures you that you are working together.
    13. Suggests solutions to the financial burden and explains the mutual benefits.
    14. **GENERAL**: Demonstrates confidence and professionalism.
    15. Avoids upward intonation at the end of statements (so it sounds like a true statement, and NOT a question).

1. **Questions to ask throughout the encounter:**
   1. **PHASE 1**: Why is this important?
   2. Why should I dedicate funding and resources to this problem?
   3. How does this help our patients or our hospital?
   4. What information do you have to support this idea?
   5. **PHASE 2**: Why are you qualified to lead this?
   6. You think you are or you KNOW you are qualified?
   7. Are you confident in your capabilities? If not, it hard for me to be confident.
   8. Do you have any data that you can share to support your qualifications?
   9. **PHASES 3 and 4:** What is your solution?
   10. What do you need to make this happen?
   11. What data/information do you have to support your request?
   12. Has this solution been used before? How did that go?
   13. What were you thinking burdening us with this cost?
   14. So you think the hospital’s experienced ICU physicians don’t know how to place a central line?
   15. So you think the high central line infection rate is OUR fault?
   16. What do you need to make this happen?
   17. Well, you don’t understand that I don’t just have this amount of money to give away. We have valuable financial obligations already that are important.
   18. What is the benefit for my division?
   19. What are the next steps?
2. **Challenges the Department Chair will present to the learner:**
   1. **PHASE 1**: As this is a cost and resource intensive request, you are hesitant to support the proposed idea. You are unaware of just how high the mortality rates and costs burdens are for CLABSI/central line infection. You are doubtful of the proposed solution for simulation based medical education without being convinced of its true value and effectiveness.
   2. **PHASE 2**: As this is a large task, you have doubts that this young, woman physician is experienced enough for such a role. You also are concerned about maintaining your dependable reputation as a physician leader and appropriate role delegation.
   3. **PHASES 3 and 4**: You do not want to be taken advantage of. You have a fixed division budget and cannot guarantee additional funds without further investigation.
   4. You are defensive that you are being asked to have your experienced staff learn central line placement, a skill that they already know. You are also worried about being able to fund projects (grand round speakers, CME accreditation, staff family picnic, research, conference sponsorship) that are aligned with your department’s priorities (patient safety, staff education, staff safety and wellness).

**Guide for the items that the resident should address:**

**PHASE 1, ELEVATOR PITCH**: Effectively share an elevator pitch in under 2 minutes and conclude with a final ask for follow up.

- Maintains a professional demeanor and displays confidence.
- States clearly the problem and why it is important.
- Explains the proposed solution.
- Uses data and provides support for her proposal.
- Communicates a clear request, deadline, and follow up.

**PHASE 2, SELF PROMOTION**: Confidently states clearly their qualifications.

- States explicitly that they are qualified and capable.
- Provides data to support their qualifications: their resume, CV, can send an email or obtain copies of their patient satisfaction scores, program feedback, etc.

**PHASE 3 and 4, CONTRACT NEGOTIAION AND DE-ESCALATION:**

- Assesses your initial offer before making their request.
- Pauses after making their request.
- Provides data to support their requests.
- Demonstrates empathy, avoids blame, and de-escalates the frustrated party.
- Determines the conflicting party’s priorities, and if there are any in common.
- Suggests mutually beneficial solutions based on common priorities.
- Maintains a professional demeanor and displays confidence.
- Summarizes negotiated agreement and necessity to obtain in writing and states the next steps.

**Pre-Post Case**

**Structure**

**Learner:** 1 - a senior woman resident or fellow in the last 1-2 years of training

**Standardized Patient**: 1 – Hospital Division Chief of Patient Safety and Quality Control (PSQC). Manages each department’s needs as well as the hospital as a whole.

**Faculty Educator**: None, no formal debrief.

**Simulation Format**: Summative simulation. Videotaped scenario for evaluation.
